# Complex Movements, Philopatry and Expanded Depth Range of a Severely Threatened Pelagic Shark, the Oceanic Whitetip (*Carcharhinus longimanus*) in the Western North Atlantic

**DOI:** 10.1371/journal.pone.0056588

**Published:** 2013-02-20

**Authors:** Lucy A. Howey-Jordan, Edward J. Brooks, Debra L. Abercrombie, Lance K. B. Jordan, Annabelle Brooks, Sean Williams, Emily Gospodarczyk, Demian D. Chapman

**Affiliations:** 1 Microwave Telemetry, Inc., Columbia, Maryland, United States of America; 2 Shark Research and Conservation Program, Cape Eleuthera Institute, Eleuthera, The Bahamas; 3 Abercrombie & Fish, Miller Place, New York, United States of America; 4 School of Marine and Atmospheric Science & Institute for Ocean Conservation Science, Stony Brook University, Stony Brook, New York, United States of America; Bangor University, United Kingdom

## Abstract

Oceanic whitetip sharks (*Carcharhinus longimanus*) have recently been targeted for conservation in the western North Atlantic following severe declines in abundance. Pop-up satellite archival tags were applied to 11 mature oceanic whitetips (10 females, 1 male) near Cat Island in the central Bahamas 1–8 May 2011 to provide information about the horizontal and vertical movements of this species. Another large female was opportunistically tagged in the U.S. Exclusive Economic Zone (EEZ). Data from 1,563 total tracking days and 1,142,598 combined depth and temperature readings were obtained. Sharks tagged at Cat Island stayed within 500 km of the tagging site for ∼30 days before dispersing across 16,422 km^2^ of the western North Atlantic. Maximum individual displacement from the tagging site ranged from 290–1940 km after times at liberty from 30–245 days, with individuals moving to several different destinations (the northern Lesser Antilles, the northern Bahamas, and north of the Windward Passage). Many sharks returned to The Bahamas after ∼150 days. Estimated residency times within The Bahamas EEZ, where longlining and commercial trade of sharks is illegal, were generally high (mean = 68.2% of time). Sharks spent 99.7% of their time shallower than 200 m and did not exhibit differences in day and night mean depths. There was a positive correlation between daily sea surface temperature and mean depth occupied, suggesting possible behavioral thermoregulation. All individuals made short duration (mean = 13.06 minutes) dives into the mesopelagic zone (down to 1082 m and 7.75°C), which occurred significantly more often at night. Ascent rates during these dives were significantly slower than descent rates, suggesting that these dives are for foraging. The sharks tracked appear to be most vulnerable to pelagic fishing gear deployed from 0–125 m depths, which they may encounter from June to October after leaving the protected waters of The Bahamas EEZ.

## Introduction

The oceanic whitetip shark, *Carcharhinus longimanus*, is a circumtropical pelagic apex predator that is poorly studied compared to many other large sharks [1]–[3]. It is thought to primarily occupy the upper layer of the water column, tolerating temperatures from 18–28°C but preferring >20°C [1]. Oceanic whitetips were historically abundant, replacing primarily temperate-dwelling blue sharks (*Prionace glauca*) as the numerically dominant pelagic shark at lower latitudes [2]. Several studies have shown substantial population declines in oceanic whitetips, most likely related to mortality associated with the global shark fin trade [4]–[6]. This species is now listed as “Critically Endangered” in the Northwest Atlantic and “Vulnerable” globally by the International Union for the Conservation of Nature (IUCN) [7]. There is growing international interest in improving the conservation of this species, including an unsuccessful proposal by the United States to add them to Appendix II of the Convention on International Trade in Endangered Species (CITES) in 2010. Among Regional Fisheries Management Organizations (RFMOs) the International Commission for the Conservation of Atlantic Tuna (ICCAT), the Inter-American Tropical Tuna Commission (IATTC) and the Western and Central Pacific Fisheries Commission (WCPFC) have prohibited the landing of oceanic whitetips by member nations.

Despite growing conservation concern, very little is known about the movements and habitat use of oceanic whitetips in the Atlantic. From 1962–1997 only 73 oceanic whitetips were conventionally tagged and just 4 recaptured as part of the U.S. National Marine Fisheries Service Cooperative Shark Tagging Program, too few to elucidate any migratory patterns (although the distances between tagging and recapture location were as high as 2,811 km [8]). Pop-up satellite archival tags (PSATs) offer a means to collect movement information from a higher fraction of tagged individuals than conventional tags [9]–[11]. PSATs have revealed hitherto unknown migrations [9] and expanded the known thermal [10] and vertical ranges [11] of other marine species, in some cases prompting reconsideration of their ecological niches or a rethinking of conservation strategies. To date, relatively few oceanic whitetips have been fitted with PSATs. Thirteen individuals tracked using PSATs in the central Pacific Ocean stayed far from land, moved long distances (140 to >4,000 km after times at liberty ranging from 10–243 days) and were strongly attached to the upper ∼ 120 m of the water column, where temperatures closely resembled sea surface temperature (>25°C) [12]. To our knowledge only one oceanic whitetip has been successfully tracked with a PSAT in the Atlantic, a 148 cm fork length (FL) male tagged in the Gulf of Mexico [13]. This individual generally exhibited a similar temperature and vertical range to conspecifics tracked in the Pacific [12], except that it occasionally made dives to depths of 150–256 m [13]. It is currently unknown if this large vertical range is typical of (or unique to) oceanic whitetips in the Atlantic.

Here, we report on the deployment of PSATs on mature oceanic whitetips (N = 12, 11 females and 1 male) in the western North Atlantic. Our objectives were to locate high-use areas, describe horizontal movements, determine if individuals were philopatric (i.e., returned to the tagging area if they left it) and characterize the patterns of vertical and thermal habitat use.

## Methods

### Ethics Statement

All research was carried out under the Cape Eleuthera Institute (CEI) research permit (MAF/FIS/17 & MAF/FIS/34) issued by the Bahamian Department of Marine Resources in accordance with CEI animal care protocols developed within the guidelines of the Association for the Study of Animal Behaviour and the Animal Behavior Society [14].

### Shark Capture and Handling

**Figure 1:**
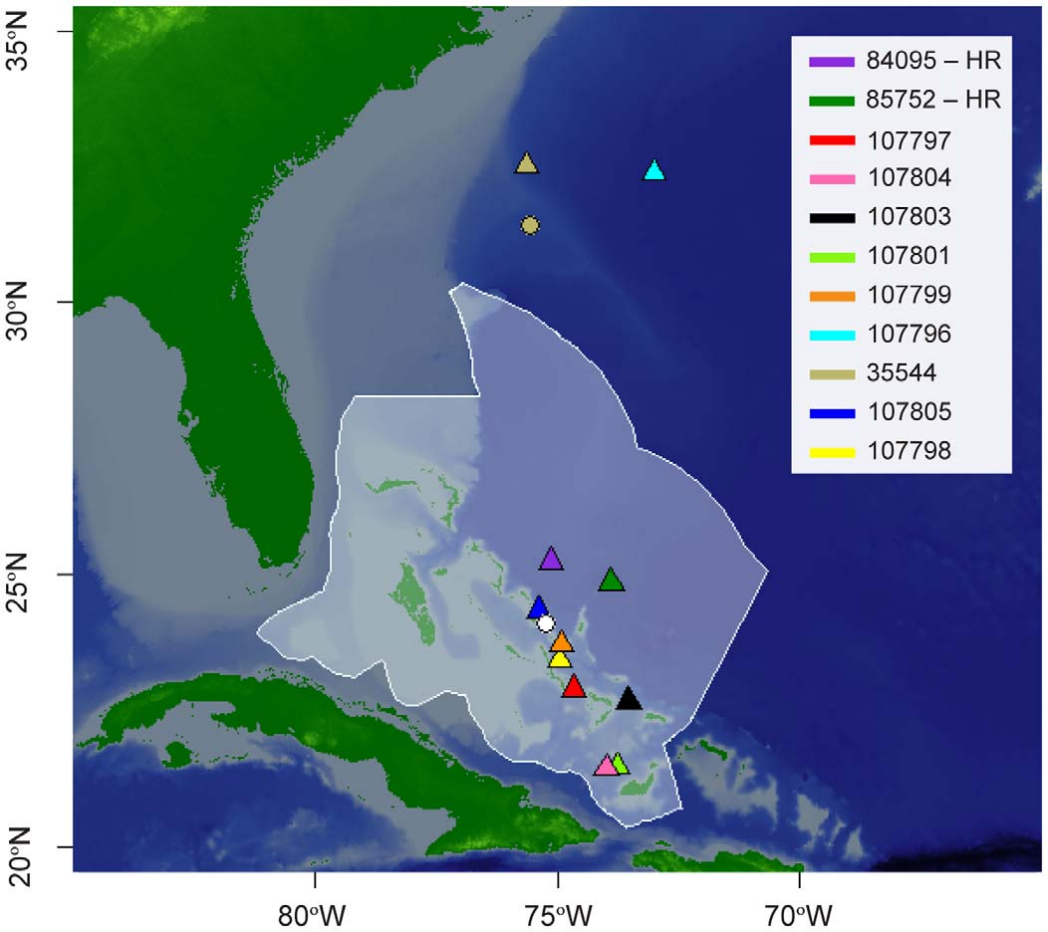
The white circle denotes the tagging location of tags deployed within the Bahamian EEZ and the beige circle denotes the tagging location of the single tag deployed outside the Bahamian EEZ. Color-coded triangles indicate pop-up location for individuals (listed by tag ID). The Bahamian EEZ is outlined in white, with white transparent fill.

After consultation with fishermen, dive tour operators and scientists, we focused tagging efforts at Cat Island, The Bahamas, the only place in the region where it appeared that a short, targeted tagging expedition was likely to be successful. Tagging was conducted from 1–8 May 2011, within 20 km of Columbus Point (24.12°N, 75.28°W; Fig. 1).

Oceanic whitetips were attracted to the research vessel using a chum crate containing fresh pieces of dolphinfish (*Coryphaena hippurus*) and Atlantic bonito (*Sarda sarda*). A few were opportunistically encountered during trolling activities, usually after a fish was hooked. Once the oceanic whitetips were sighted, baited hand-lines were deployed, consisting of 8 m of nylon line (6 mm diameter) with one large float (37 cm diameter) attached at one end, an 18/0 non-offset circle hook with approximately 80 cm of steel leader attached to the other end, and a second smaller float (20 cm diameter) attached to the line approximately 1.5 m from the leader to provide additional flotation to the gear. Once hooked and brought alongside the vessel, all sharks were measured and their sex visually determined by the presence or absence of claspers (present in males). Two uniquely numbered external tags were deployed on all captured sharks: a Rototag® attached to the upper third of the first dorsal fin (Dalton Tags, Henley-on-Thames, United Kingdom), and a stainless steel M-type dart tag inserted in the basolateral dorsal musculature (Hallprint, Victoria Harbour, Australia). PSATs were inserted into the dorsal musculature lateral to the first dorsal fin with a handheld tagging applicator. The tag was anchored with a plastic umbrella dart [15] and trailed from 19 cm of 220-lb test monofilament leader secured with stainless steel crimps and encased in surgical silicone tubing. Hooks were completely removed by cutting the barb and rotating the hook free immediately prior to release. One PSAT was given to a U.S. swordfish longline vessel to deploy on an incidentally captured oceanic whitetip.

### Satellite Tag Details

X-Tags (Microwave Telemetry, Inc., Columbia, MD, USA) were used for this study. X-Tags are 12×3.2 cm (excluding antenna) and weigh 40 g in air. At a pre-programmed pop-up date (between 30–245 days for this study) an electrical current sent through a corrodible link causes the tags to detach from their tethers. Tags are then free to float to the surface and begin transmitting data through the Argos satellite system. X-Tags are also programmed to detach under conditions of constant pressure (depth) recorded over a specific time period (4 days) or after reaching a depth where the physical integrity of the tag may be compromised (manufacturer specified at 1250 m). Standard Rate (SR) X-Tags recorded temperature, depth, and light level at two-minute intervals. Because of battery and Argos system data throughput limitations, a subset of the recorded (archived) data is transmitted through the satellite system during the transmission period (18.3±1.60 SE days in the current study). The temporal resolution of these data depends on deployment duration. For deployments less than four months, 15-minute readings were transmitted. For deployments over four months but less than eight months, the 15-minute readings were overwritten with 30-minute readings. For deployments over eight months, the 30-minute readings were overwritten with hourly readings. Daily sunrise and sunset times were calculated onboard the SR tag using a proprietary algorithm. Daily geolocations were calculated from the transmitted daily sunrise and sunset times. High Rate (HR) X-Tags set for 30-day deployments recorded depth and temperature at five-minute intervals. Temporal resolution of transmitted data is also at five-minute intervals. While HR tags do record low-resolution light levels, they do not calculate sunrise and sunset times. Tag recovery allows access to the entire two-minute recorded (archived) data in the case of a SR tag and the entire five-minute recorded data of a HR tag (http://www.microwavetelemetry.com/fish).

### Data Treatment and Statistical Analysis

#### Horizontal movements

A state-space unscented Kalman filter with sea surface temperature (UKFSST) was used on all available geolocations to estimate positions and corresponding 95% confidence intervals from SR tags [16]. HR tags do not provide enough light data for geolocation estimation and were excluded from movement analyses, except for reporting the linear distance between tagging and pop-off location. The NOAA Optimum Interpolation Sea Surface Temperature V2 dataset provided weekly SSTs on a one-degree (latitude/longitude) grid (http://www.esrl.noaa.gov/psd/). The daily SST needed for geolocation estimation was identified as the daily maximum temperature transmitted on a given day. For days without a transmitted maximum temperature, local polynomial smoothing was applied to estimate the missing values. UKFSST does not use bathymetry in position estimation. Therefore, an additional bathymetric correction was applied to the UKFSST position estimates that violated known bathymetric data (such as position estimates on land). The correction involved randomly sampling positions within the confidence intervals and subsequently comparing tag daily maximum depths to known bathymetric data. For days when depth records were not transmitted through the Argos system, the maximum depth was considered to be zero (i.e., at the surface). The bathymetric dataset has a one-minute resolution (http://coastwatch.pfeg.noaa.gov). This analysis was completed using the analyzepsat package for R [17], [18]. The linear distance between the tagging location and the daily estimated positions and the linear distance between each consecutive estimated position were determined with the geosphere package in R [19]. In order to generate the utilization distribution, individual probability densities for each UKFSST estimated position were determined from the positions' corresponding variance. All individual probability densities from sharks tagged in The Bahamas were combined to create a collective probability density represented as a percent volume. This calculation was completed on a 0.1° by 0.1° (Latitude/Longitude) grid in the region encompassing the tracks (80°W, 55°W, 15°N, and 45°N). The analyzepsat package in R was used for this analysis [17], [18]. The Bahamas Exclusive Economic Zone was identified from the coordinates extracted from a kmz file retrieved from VLIZ Maritime Boundaries Geodatabase (http://www.vliz.be/vmdcdata/marbound/index.php). The estimated percentage of time in the Bahamian EEZ was calculated for each individual (excluding tag 35544) by taking the ratio of UKFSST estimated positions inside and outside the EEZ boundary. Days missing geolocations were not included in the determination of residency times. The tag transmits data in a pseudo-random order so that any missing data are randomly distributed throughout the deployment duration (http://www.microwavetelemetry.com/fish/Xtag.cfm).

#### Vertical movements

The tags implement data compression techniques prior to transmission, and as a result, selected depth and temperature values in SR datasets may be identified as delta limited. A depth record marked as a delta limited dive may actually be deeper than the transmitted value while a temperature record marked as a delta limited decrease may actually be colder than the transmitted value. Similarly, a depth (or temperature) record marked as a delta limited ascent (increase) may actually be shallower (warmer) than the transmitted value (http://www.microwavetelemetry.com/fish/understanding_data_xtag.cfm). Delta limited temperature values comprised 0.07% of the complete temperature dataset, and delta limited depth values comprised 0.67% of the complete depth dataset. All delta limited values were included in the analyses unless otherwise noted. The depth resolution of SR X-Tags ranges from 0.34 m –5.4 m. Physically recovered SR X-Tags have a constant depth resolution of 0.34 m, and HR X-Tag depth resolution is 1.34 m. The temperature resolution for all tags in the study ranges from 0.16–0.23°C (http://www.microwavetelemetry.com/fish/).

The thermocline base resembles the 20°C isotherm topography [20]. The depth corresponding to the 20°C isotherm in our dataset was identified by analyzing concurrently recorded depth-temperature pairs (N = 557971; excluding delta limited values). The mean temperature at each depth was determined, and the minimum depth with a corresponding mean temperature ≤20°C was identified as 204 m. For practical purposes, the depth of the 20°C isotherm was denoted as 200 m in the subsequent statistical analysis. Additionally, this depth threshold marks the upper boundary of the mesopelagic zone. For transmitted datasets, a dive was defined as a depth record below the 200 m isobath. Datasets from physically recovered tags have increased temporal resolution (two minutes), and therefore, a single dive was defined as a series of consecutive depth records below 200 m. Recovered tags allowed for the estimation of dive descent and ascent rates using the archived data. The dive descent included the depth records from the start of the dive (i.e., first depth record above 200 m, immediately before descending below the 200 m isobath) to the deepest depth in the dive. The dive ascent included depth records from the deepest depth to the first depth record above the 200 m isobath. Average dive descent and ascent rates were generated from the length (m) of the descent (or ascent) divided by the time duration of the descent (or ascent). These data significantly differed from a normal distribution (Shapiro-Wilk test). Thus, prior to analysis, datasets were transformed (log_10_[x +1]), and a t-test was used to determine statistical significance between descent and ascent rates.

Diel periods (i.e., diurnal, dusk, nocturnal, and dawn) were determined from the sunrise and sunset times at Cat Island (24.12°N, 75.28°W) (http://aa.usno.navy.mil/). The dawn period was defined as one hour before and one hour after mean monthly sunrise, while dusk was considered one hour before and after mean monthly sunset. Diurnal and nocturnal periods were represented by the time following dawn and dusk, respectively. All depth and temperature records were divided into groups based on diel periods. Both depth and temperature exhibited non-normal distributions, and consequently these datasets were Box-Cox transformed (temperature λ = 4, depth λ = 0.46) prior to analysis. Daily mean depths and temperatures in each diel period, for each individual, were calculated. Crepuscular periods (dawn and dusk) were omitted from the analysis; only day and night were compared for differences. Additionally, the means from the day and night periods on the first deployment day for each tag were omitted from the analysis. Differences in depth and temperature means between periods were evaluated independently with linear mixed effects models [21]–[22]. A repeated measures form of the following model was used for comparison [23]:
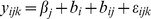
(1)


*β_j_* = the fixed effect of diel period j;

*b_i_* = the random effect due to individual shark (*b_i_* ∼ iid *N*(0, σ_1_^2^));

*b_ij_* = the random effect of each diel period within each individual shark (*b_ij_* ∼ iid *N*(0, σ_2_^2^));

*ε_ijk_* = error terms (*ε_ijk_* ∼ iid *N*(0, σ_ε_^2^)).

The repeated measures form of Equation (1) includes a covariance structure to account for the within-individual autocorrelation. In order to determine the most appropriate form, potential covariance structures were fitted to the data and evaluated based on Akaike's information criterion (AIC) [21]. The autoregressive moving average (ARMA) covariance structure was the best fitting model for both depth and temperature (depth autoregressive order, p = 1; depth moving average order, q = 1; temperature autoregressive order, p = 2; temperature moving average order, q = 1). From an initial analysis of the data, individuals appeared to behave differently during diel periods, and therefore, an interaction term was included in the model. Based on the likelihood ratio test, the model including the interaction term was a better fit for both depth and temperature comparisons (p<0.001) [23]. Model parameters were estimated with restricted maximum likelihood. This analysis was completed with the nlme package in R [24].

To examine diving periodicity below the 200 m isobath, diving frequency was standardized. This was achieved by counting the number of dives per day in both diel periods (day and night), for each individual, and dividing that value by the number of records in the corresponding period that were above the thermocline base (200 m). These data were non-normal and transformation attempts did not adequately improve distributions for use with parametric analysis. Therefore, the comparison of diving frequency between diel periods was evaluated with a Mann-Whitney test.

Daily mean depths for each individual were determined and time-paired with the UKFSST estimated SST values from the NOAA Optimum Interpolation Sea Surface Temperature V2 dataset. Any dates without both depth data and UKFSST positions were omitted. The mean depths were log-transformed. In order to detect a relationship between mean depth and SST, Pearson's product-moment correlation test was applied to the log-transformed daily mean depths versus daily SST. All analyses were completed in R [19].

## Results

### General

**Figure 2:**
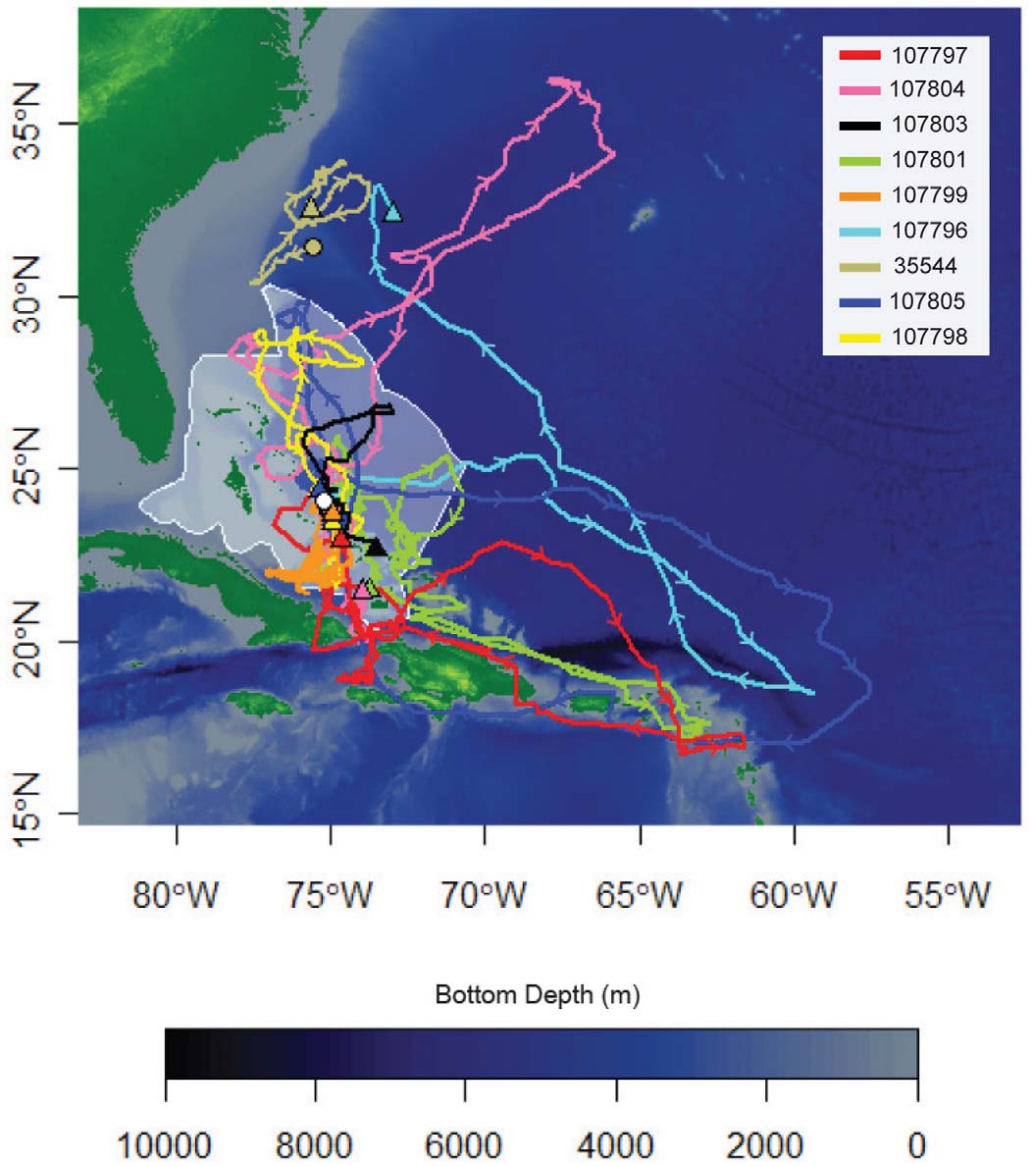
Map with bottom depth (m) showing filtered tracks for nine oceanic whitetip sharks equipped with Standard Rate tags. Colored lines represent tracks from individuals (listed by tag ID); triangle indicate pop-up location. Arrows on colored lines show direction of movement.

**Figure 3:**
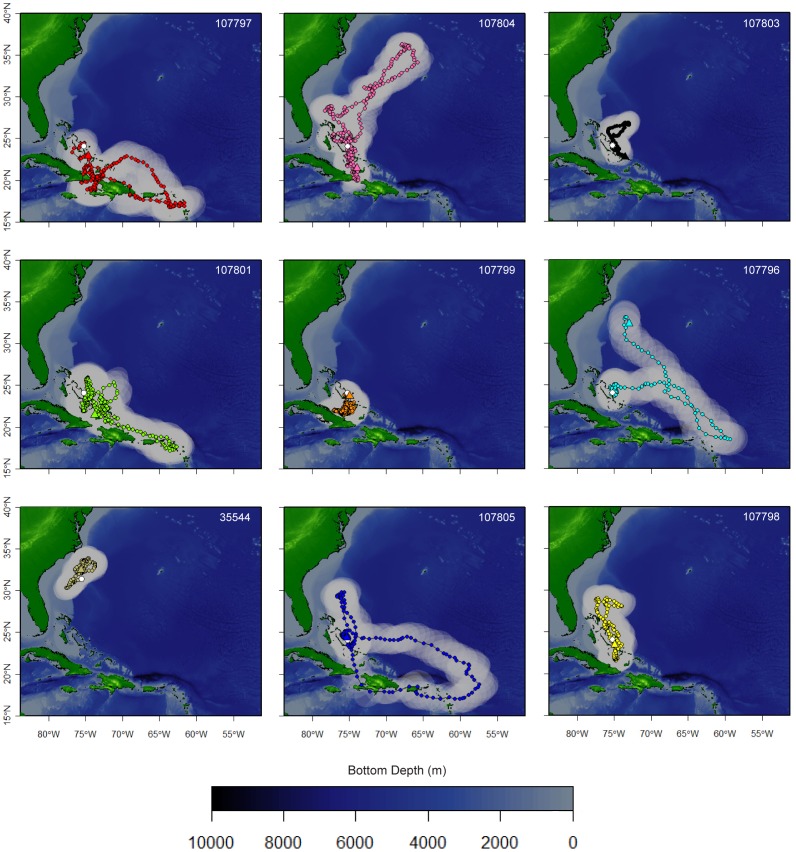
Map showing tracks of nine individuals tagged with Standard Rate X-Tags,including 95% confidence intervals (translucent ellipses) based on UKFSST. Color-coded dots indicate daily estimated geolocations for nine individuals (listed by tag ID).

One male and 10 female oceanic whitetips were tagged from May 1–8, 2011 near Cat Island, nine with SR tags and two with HR tags (Table 1). Another female was opportunistically fitted with a SR tag from a commercial longline vessel operating off the U.S. continental shelf (31.44°N, 75.56°W). Although the pop-off location and estimated track of this individual is depicted (Figs. 1, 2, 3), its track information was not included in all analyses involving pooled data from sharks tagged in The Bahamas. Instrumented sharks ranged in length from 207–285 cm (TL), which exceeds the size at maturity established in other studies [1]–[3]. The male had long, calcified claspers, also indicative of sexual maturity.

**Table 1:** Summary of biological and tag information from all tagged sharks.

Tag ID	Sex	Fork Length(cm)	Date Deployed(M/D/Yr)	Pop-offDate	Days at Liberty	Pop-off Latitude(°N)	Pop-off Longitude(°W)	Data Received(%)	Mean (± SE)Depth (m)	Mean (± SE)Temperature (°C)	Maximum Depth (m)	Minimum Temperature (°C)
84095^a^	F	193	5/5/11	6/5/11	31	25.244	75.138	81	47.62 (±0.49)	25.17 (±0.01)	441	18.26
85752^a^	F	204	5/5/11	6/5/11	31	24.867	73.893	82	44.63 (±0.37)	25.03 (±0.01)	265	19.55
107797^b^	F	218	5/1/11	11/1/11	184	22.889	74.679	100	59.95 (±0.12)	26.35 (±0.0046)	1082	7.75
107804	F	161	5/8/11	1/8/12	245	21.445	73.986	49	31.48 (±0.35)	26.64 (±0.02)	500	17.62
107802	M	207	5/6/11	N/A	N/A	N/A	N/A	N/A	N/A	N/A	N/A	N/A
107803	F	165	5/7/11	8/7/11	92	22.691	73.555	68	34.09 (±0.33)	26.39 (±0.02)	382	18.58
107801	F	213	5/5/11	1/5/12	245	21.477	73.772	39	61.55 (±0.65)	26.06 (±0.03)	925	9.16
107799^b^	F	214	5/4/11	1/4/12	245	23.727	74.934	100	50.08 (±0.09)	26.59 (±0.0035)	752	10.89
107796	F	212	5/1/11	8/1/11	92	32.381	72.996	77	38.02 (±0.46)	26.14 (±0.02)	624	16.67
35544^c^	F	183^d^	5/2/11	8/2/11	92	32.509	75.633	65	39.40 (±0.40)	25.66 (±0.02)	742	13.69
107805^b^	F	233	5/1/11	10/1/11	153	24.363	75.365	100	41.72 (±0.11)	25.98 (±0.0050)	720	7.9
107798^b^	F	189	5/4/11	10/4/11	153	23.201	74.914	100	46.15 (±0.12)	26.16 (±0.0057)	1008	8.54

a high-rate tag.

b recovered tag.

c individual tagged at 31.439°N 75.562°W on commercial longliner.

d estimated total length.

Eleven of 12 tags reported data through the Argos system (Table 1). The SR tag affixed to the male shark never reported (107802). All reporting tags remained attached and reached their pre-programmed pop-off dates. The two HR tags deployed for 30 days (84095, 85752) popped-off in the central Bahamas, 125 and 163 km from Cat Island, respectively. Tag 35544 popped-off 119 km from the its tagging site in the U.S. EEZ in August. Also in August, tag 107796 popped-off ∼ 760 km from Bermuda after a 92-day deployment, 946 km to the northeast of Cat Island. The remaining tags all popped-off in The Bahamas (Fig. 1), 28–331 km from Cat Island (mean = 172 km) after deployment durations of 92 (1 tag), 153 (2 tags), 184 (1 tag), and 245 days (3 tags). Four SR tags were physically recovered, allowing for extraction of their complete archival high-resolution (two-minute) datasets. For the seven tags from which transmitted datasets were obtained, data reception ranged between 39–82% (Table 1). In total, 1494 geolocation days, 571,496 depth readings, and 571,102 temperature records were used for analysis. All the SR tags reported 100% of geolocation days except 107801 and 107804 which were missing five and two days, respectively.

### Horizontal Movements

**Figure 4:**
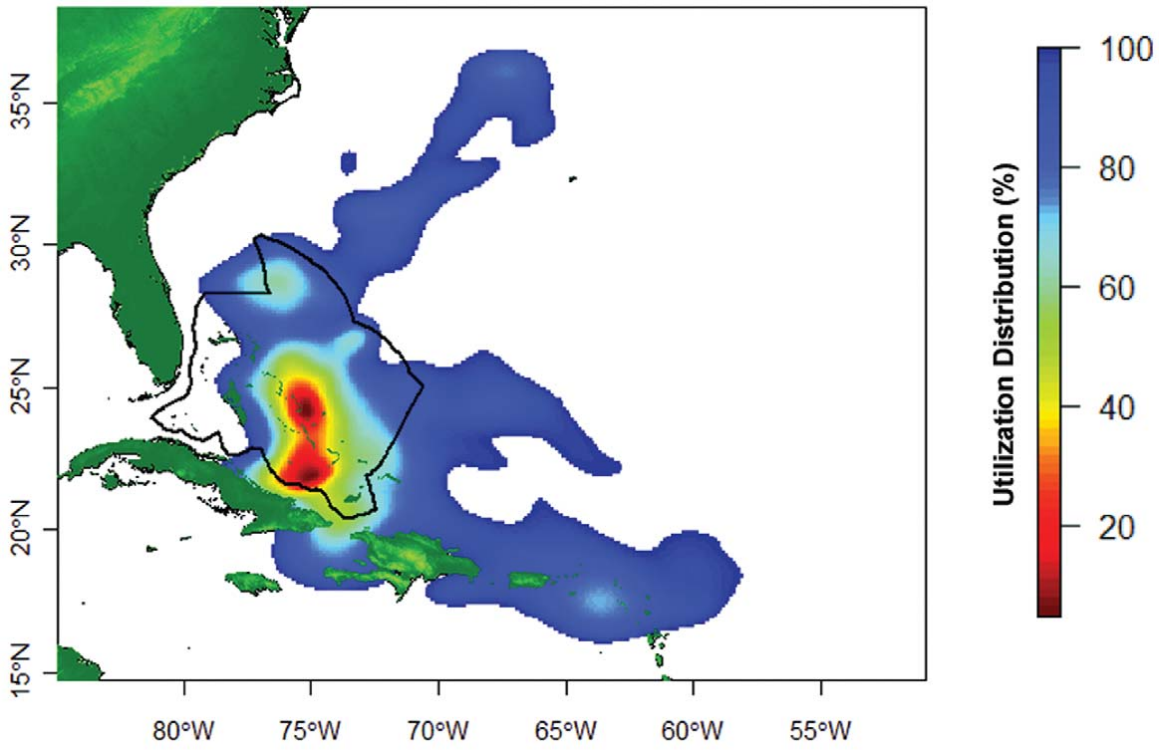
Map showing percent utilization distribution calculated from filtered daily geolocations from the eight individuals equipped with Standard Rate tags deployed at Cat Island, The Bahamas. Black line indicates the boundary of the Bahamian EEZ.

The 95% utilization distribution area for all tagged sharks during the study period was 16,422.11 km^2^ (Figs. 3–4). This was derived using data from sharks originally tagged within an area of ∼ 20 km^2^, excluding the shark tagged outside of The Bahamas. The highest density of geolocation estimates within this area occurred between Ragged Island (22.08°N, 75.41°W) and Cat Island, all within the boundary of The Bahamas EEZ (Fig. 4). Other relatively high-use locations include: the Leeward Islands (northern Lesser Antilles), the northern Bahamas, and north of the Windward Passage; which are approximately 1400 km, 600 km, and 450 km from the Cat Island tagging site, respectively (Fig. 4).

**Figure 5:**
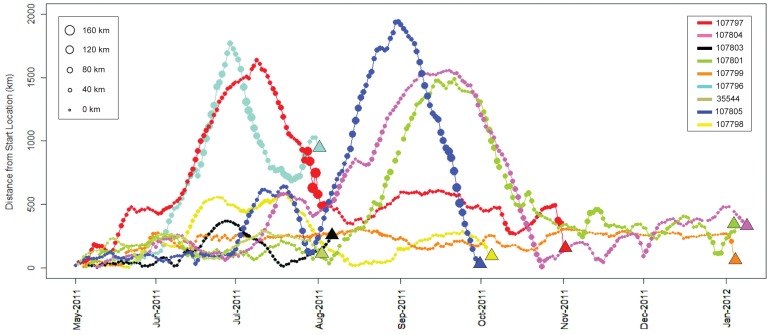
Chart showing distance from start location (km) by date for nine oceanic whitetip sharks equipped with Standard Rate tags. Circles of varying size indicate distance traveled (km; five-day central moving average) during periods of the track. Triangles represent distance from tagging site to pop-up location. Colors correspond to tag ID.

All of the tagged oceanic whitetips remained within 500 km of their tagging location for the first 30 days of their tracks (Figs. 2, 5). At least some individuals remained in close proximity to Cat Island during this period based on reported observations of tagged sharks from local dive operators and the nearby pop-off locations of the two HR tags (Fig. 1, Table 1). Of the eight remaining sharks, two (107796, 107797) made relatively rapid southeasterly movements, traveling >1500 km from Cat Island within 50–75 days of tagging (i.e., in late June and July; Figs. 2, 5). One of these (107796) then looped to the northwest towards Bermuda, where the tag popped-off. Three other individuals moved away from the central Bahamas at a slower rate, but eventually traveled >1,500 km from Cat Island after 100–150 days (Figs. 2, 5). Two of these individuals (107805, 107801) moved to the southeast (although one initially went north), while the other one moved northeast towards Bermuda (107804). The remaining three sharks stayed within 700 km of the tagging site for their entire track, staying inside or very near The Bahamas EEZ (Figs. 2, 5). Four of the five individuals that made long-distance movements began moving back towards The Bahamas later into their tracks (Figs. 2, 5). Individuals still carrying tags after 150 days (October/November) were all located within 500 km of Cat Island when their tags popped-off (Figs. 1, 5). One of the sharks tagged with a HR tag in 2011 (85752) was later photographed at Cat Island on 24 April 2012. Estimated percent time spent in the Bahamian EEZ ranged from 24–100% with a mean of 68.2% ±9.3 SE.

### Vertical Movements and Temperature Range

**Figure 6:**
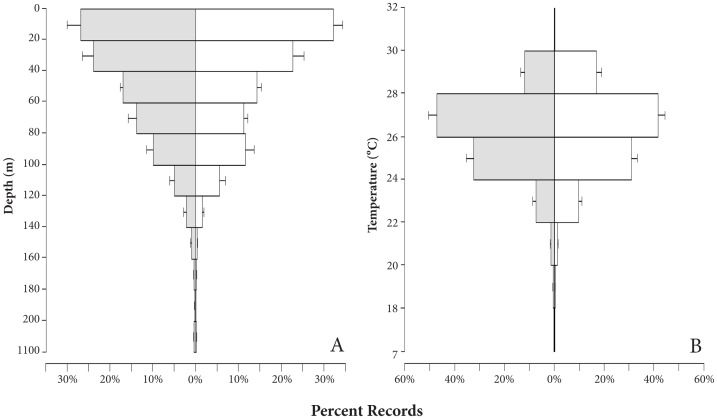
Percent records for night (gray) and day (white) for 11 tracked individuals. Mean (± SE) percent records for A) depth (m) and B) temperature (°C) for all tagged sharks.

**Figure 7:**
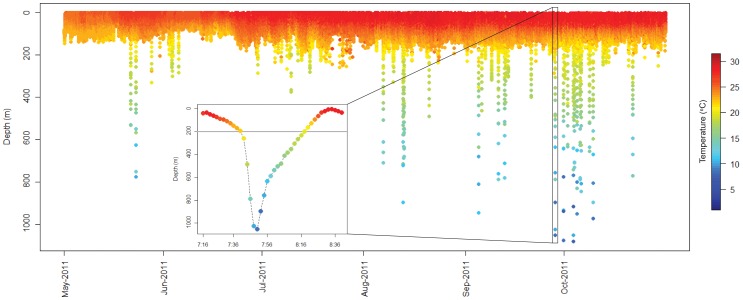
Chart of time-series depth (m) data with time-paired temperature (°C) data from a recovered tag (107797; two-minute data intervals) deployed for six months. Inset depicts an example of a typical dive profile.

Sharks generally associated with the epipelagic zone (0–200 m) throughout their track, with 99.7% of all depth records within this range (Fig. 6A). Mean (± SE) depth was 49.39 (±0.05) and the maximum depth recorded was 1082 m. Temperature records ranged from 7.75–30.48°C, with a mean of 26.27°C (±0.002) (Fig. 6B). The lowest temperatures occurred when sharks made dives below the thermocline (i.e., 200 m, Fig. 7). The number of dives below the thermocline ranged from 0.06–0.66 per day (mean = 0.37±0.06). Based on data from the four recovered tags, the mean maximum depth of dives below the thermocline was 323.4 m (±9.07). The mean duration of these dives was 13.06 min (±0.57, N = 351) and, during these dives, individuals descended significantly faster (mean = 37.3 m/min ±1.48) than they ascended (mean = 17.1 m/min ±0.49) (t = 11.76, p<0.001).

### Diel Depth/Temperature Use

The linear mixed effects models indicated no significant differences between mean depth and mean temperature utilization during day and night diel periods (Table 2, Fig. 6). Time-paired depth and temperature records exhibited an exponential relationship (p<0.001, r = −0.66, r^2^ = 0.44). Examination of diving activity (i.e., dives below 200 m) showed that these deep dives were significantly more common at night (W = 1040551, p<0.001; Table 2). Additionally, a Pearson's test revealed a significant correlation (p<0.001, r = 0.44) between mean daily depth and mean daily SST (based on UKFSST for corresponding day). However, the mean daily depth only explained 19% of the variation of mean daily SST.

**Table 2:** Night and day mean (± SE) depth (m), temperature (°C), and number of dives (>200 m) record^−1^ period^−1^.

	Night	Day	p-value
Depth (m)	52.10±0.08	48.38±0.08	0.6546
Temperature (°C)	26.23±0.003	26.26±0.003	0.8057
Dives record^−1^period^−1^	0.00143±0.00030	0.00086±0.00017	**2.763×10^−6^**

Depth and Temperature p-values based on repeated measures linear mixed effects models. Bold p-values indicating significant difference.

## Discussion

PSATs proved to be an effective tool for tracking the movements and habitat use of mature female oceanic whitetips. Our retention-to-term and report rate rank among the highest recorded in any shark satellite tagging study (12 of 13 tags) [25]. In addition, we were able to recover a high proportion of the tags once they washed ashore in The Bahamas.

### Horizontal Movements

Five out of eight of the tagged oceanic whitetips tracked in The Bahamas for more than 31 days made long-distance movements after an initial ∼ 30-day period of residency within ∼ 500 km of the tagging area. This residency period in the central Bahamas corresponds to anecdotal reports from local scuba diving operators as to when the species is locally abundant. After this period sharks became spatially disaggregated, with individuals moving along one of several different trajectories away from the central Bahamas. Ultimately, tagged individuals moved maximum linear distances ranging from 290.36–1939.88 km from their tagging location and made overall movements of 1810.01–7941.08 km (i.e., the total length of the track). This compares well with tag-recaptures in the Atlantic, previous satellite tracking in the Pacific, and further highlights the highly migratory nature of at least some oceanic whitetips.

Mature female oceanic whitetips tagged at Cat Island were not uniform in their movement patterns in the months after they were tagged. A surprisingly high fraction (3 of 8) of the sharks tracked for >31 days stayed within or very near The Bahamas EEZ for their entire track. The remaining sharks made long-distance movements outside of the EEZ. The variability in movements and dispersal range observed in mature female oceanic whitetips in this region is unlike other pelagic animals in the North Atlantic (e.g., blue sharks, bluefin tuna, porbeagle sharks [18], [26]–[27]), where movement patterns are generally more uniform within a single demographic group tagged in the same area.

The range of movement patterns observed in this study may be attributable to differences between individuals in particular stages of the reproductive cycle. Oceanic whitetips are believed to have a biennial reproductive cycle, giving birth on alternate years [1]–[3], [28]), which suggests that some of the differences in individual movements may correspond to migrations by gravid and non-gravid females to disjunct pupping and mating areas. Mating occurs in the late summer in the Southern Hemisphere, with parturition likely taking place 12 months later [29]. If the same holds true in the Northern Hemisphere, then the late-summer destinations of these sharks (i.e., the northern Lesser Antilles, the northern Bahamas, and north of the Windward Passage) might be pupping or mating grounds for oceanic whitetips. Castro (2011) [3] reported examining “numerous small specimens 77–85 cm caught just north of The Bahamas,” a size range corresponding to young-of-the-year individuals. Backus et al. (1956 ) [28] reported that males were more common in the Windward Passage than females, which could mean this is a mating area. More research on the location of key pupping and mating areas in the region is clearly needed, but we hypothesize that the high-use areas we document in this study serve one or both of these functions for this species.

Oceanic whitetips generally exhibited maximum displacement from Cat Island 50–140 days after tagging (end of June through September), after which all but one (107796) individual headed back to the central Bahamas. All individuals tracked for longer than 90 days were 28–331 km from Cat Island when their tags popped-off, possibly indicating the beginning stages of a return to the tagging location. Supporting this, one individual (85752) was photographed by scuba divers on two occasions during 23–24 April 2012, within 6.5 km of the tagging site. “Philopatry” has previously been defined as the return of individuals to their birthplace, home range, or another adopted locality [30]. Under this definition, these mature female oceanic whitetips appear to be philopatric to the central Bahamas. This is, to our knowledge, the first evidence of philopatry in this highly mobile pelagic species. Although we are only able to speculate at this time, oceanic whitetips may return to this area because of the local abundance of large epipelagic prey, such as billfishes, tunas, and dolphinfish. There are also a small number of shark dive operators in the area that provision oceanic whitetips, but these operators provide such a small amount of food that it is unlikely this is the sole motivation for these individuals return to the area from hundreds or thousands of kilometers away.

### Vertical and Thermal Range

Oceanic whitetips primarily occupied the epipelagic zone shallower than 125 m, very similar to tagged conspecifics in the Pacific [12]. As a result, individuals spent most of their time in waters with temperatures similar to SST. This fits previous characterizations of the ecological niche of oceanic whitetips as being subtropical to tropical predators of epipelagic fishes [1]–[3], [28]. Vertical activity showed no clear diel patterns in depth and temperature range, with the exception that dives below the thermocline were more common at night.

For tagged sharks in our study, a weak relationship between mean daily depth and mean daily SST was found. As individuals experienced warmer SST, likely resulting from seasonal sea surface warming or migration to areas with warmer SST, mean daily depth increased. Behavioral thermoregulation (i.e., active avoidance or use of heat sources to regulate body temperature), has been observed in other pelagic shark species and may explain the relationship between depth and water temperature [26], [31]–[32]. However, while thermoregulatory movements could account for the weak depth and SST relationship in oceanic whitetips, other processes (e.g., prey distribution) may also influence vertical distribution [26].

All oceanic whitetips tracked made occasional dives into the mesopelagic zone, to depths of up to 1082 m with ambient temperatures as low as 7.75°C. Although similar to dives made by other pelagic sharks and teleosts [33]–[35], to our knowledge these are the deepest dives and lowest temperatures ever recorded for this species. Deep diving appears to be common in sharks, found in species ranging from active predators (e.g., salmon sharks, white sharks, etc.) to planktivores (e.g., whale sharks, basking sharks) and species living in habitats ranging from coral reef slopes (e.g., Caribbean reef sharks) to the open ocean (e.g., blue sharks) [36]–[38]. Deep diving was infrequent and brief in Atlantic oceanic whitetips, making the motivation for diving difficult to ascertain. Cephalopods have been reported as an important prey item of oceanic whitetips [1]–[3], [28]. Yet, the increase in diving during the night observed in these sharks contrasts some other pelagic species that feed on squid (e.g., blue sharks), which dive deeper at dawn and during the day, presumably following the diel vertical migration of their prey [26]. It is possible that oceanic whitetips dive to feed on mesopelagic cephalopods just as short-finned pilot whales (*Globicephala macrorhynchus*) do [39]–[40], since oceanic whitetips have been reported to associate with these cetaceans [41]. The “fast descent, slow ascent” dive profile observed in oceanic whitetips also occurs in other pelagic sharks and is postulated to be prey searching behavior [42]–[43]. All of these factors suggest that mesopelagic diving by oceanic whitetips is most likely related to foraging. Interestingly, Pacific oceanic whitetips had a shallower depth range than these Western Atlantic individuals [12]. This may be because the oxygen minimum layer is relatively deep in the western Atlantic, allowing many epipelagic fish to have a wider depth range in this region [44].

### Conservation Implications

Assuming that catchability is a function of time spent at the same depth as baited hooks, oceanic whitetips are most vulnerable to fishing gear set in the upper 125 m of the water column or to deeper-set gear while it is being deployed or hauled. A dramatic decline in oceanic whitetips catch per unit effort (CPUE) between 1950 s exploratory longline sets (mean hook depth = 72 m) and observed commercial sets in the late 1990 s (mean hook depth = 110 m) in the Gulf of Mexico has been interpreted as a regional collapse of the species [45]. The magnitude of the projected decline in the Gulf [45] has been questioned because, among other significant changes in gear, some reduction in CPUE may have occurred due to the longlines being set at greater depths in the 1990 s [46]. For comparison, 28.3% of depth records in this study occurred within the 1950 s hook depth range (53–91 m) and 19.3% were in the 1990 s hook depth range (82–138 m). This confirms that the depth distribution of both sharks and fishing gear should be considered when interpreting CPUE trends for this species [46].

Five of the sharks tagged in The Bahamas made transboundary movements, spending time in waters managed by different countries (U.S.A., Cuba, and several of the windward Caribbean islands) or the high seas that are managed by the United Nations. This illustrates why it is essential for international regulatory bodies to coordinate conservation efforts for this species across multiple jurisdictions. The total area of ocean used by this group of sharks over the study period was vast, 16,422.11 km^2^, highlighting how recently enacted landing prohibitions under ICCAT requires nations to monitor fishing activity over large areas. It is difficult to envision how countries that have limited capacity to monitor their fleets or foreign fleets fishing in their EEZ would be able to effectively enforce the landing moratorium on oceanic whitetips. For this reason, a CITES Appendix II listing could augment ICCAT prohibitions by heightening surveillance of transboundary trade, which is likely to be more centralized (i.e., airports and container shipping ports) than all of the widely distributed fishing vessels and ports where oceanic whitetips and their fins may be landed illicitly.

One unexpected outcome of tracking oceanic whitetips was the finding that individuals spent a large portion of the year in the EEZ of The Bahamas. This is attributable to several features of these tracks: (1) individuals remained within 500 km of the tagging site during May and part of June (2) limited displacement (<500 km from Cat Island) of 22.2% of tagged individuals in subsequent months and (3) the return of the individuals that left the EEZ after 60–160 days (philopatry). Pelagic longlining was prohibited by The Bahamas in the 1990 s, which may have helped conserve oceanic whitetips in this area even as they declined elsewhere in the North Atlantic [4]. The Bahamas subsequently reinforced this measure with a commercial trade ban on all sharks in 2011. These steps could be very significant for the restoration of oceanic whitetips in the region, given the extensive use of The Bahamas by mature females.
